# The Impact of Anxiety and Depression in Chronic Obstructive Pulmonary Disease

**DOI:** 10.3390/arm91020011

**Published:** 2023-03-10

**Authors:** Mandeep Singh Rahi, Bright Thilagar, Swetha Balaji, Sivaguha Yadunath Prabhakaran, Mayuri Mudgal, Suganiya Rajoo, Prashanth Reddy Yella, Palak Satija, Alsu Zagorulko, Kulothungan Gunasekaran

**Affiliations:** 1 Department of Pulmonary and Critical Care Medicine, Yale New Haven Health, Lawrence + Memorial Hospital, New London, CT 06320, USA; 2 Department of Internal Medicine, Mayo Clinic, Rochester, MN 55905, USA; 3 Division of Endocrinology and Metabolism, Department of Medicine, University of Illinois, Chicago, IL 60607, USA; 4 Department of Internal Medicine, Kasturba Medical College, Mangalore 575001, India; 5 Department of Medicine, Camden Clark Medical Center, School of Medicine, West Virginia University, Parkersburg, WV 26101, USA; 6 Department of Hematology and Oncology, WakeMed, Raleigh Campus, Raleigh, NC 27610, USA; 7 Department of Internal Medicine, Yuma Regional Medical Center, 2400 S Avenue A, Yuma, AZ 85364, USA; 8 Department of Psychiatry, Illinois Center for Neurological and Behavioral Medicine, Des Plaines, IL 60016, USA; 9 Department of Pulmonary Diseases and Critical Care, Yuma Regional Medical Center, Yuma, AZ 85364, USA

**Keywords:** chronic obstructive pulmonary disease, COPD, depression, anxiety, cognitive behavioral therapy, CBT, mental health

## Abstract

**Highlights:**

Chronic obstructive pulmonary disease (COPD) patients experience a higher level of psychological distress than the general population, including anxiety and/or depression. This article reviews the pathophysiology, clinical features, impact of mental health disorders in COPD patients. It dives into the current screening, diagnosis, and management of this population.

**What are the main findings?**

- COPD patients with comorbid anxiety or depression experience more acute exacerbations, incidences of rehospitalization, and carry a higher risk of mortality than COPD patients without these comorbidities.
- COPD patients benefit from non-pharmacological and pharmacological intervention based on severity of depression.

**What is the implication of the main findings?**

- Ongoing investigation and research are necessary to ensure appropriate screening, diagnosis, and management of COPD patient with anxiety and/or depression.
- Adequate treatment of COPD patients with comorbid mental health conditions can decrease the burden on the healthcare system by improving symptomatology and rehospitalizations.

**Abstract:**

Patients with COPD (chronic obstructive pulmonary disease) are at a higher risk of comorbid conditions such as anxiety and/or depression, which in turn increase their symptom burden and rehospitalizations compared to the general population. It is important to investigate the pathophysiology and clinical implications of mental health on patients with COPD. This review article finds that COPD patients with anxiety and/or depression have a higher rehospitalization incidence. It reviews the current screening and diagnosis methods available. There are pharmacological and non-pharmacologic interventions available for treatment of COPD patients with depression based on severity. COPD patients with mild depression benefit from pulmonary rehabilitation and cognitive behavioral therapy, whereas patients with severe or persistent depression can be treated with pharmacologic interventions.

## 1. Introduction

Chronic obstructive pulmonary disease (COPD) is a major health problem. With 3.23 million fatalities from COPD in 2019, it is the third most common cause of death globally. Nearly 90% of COPD deaths occurs in those under 70 years of age, and mostly in low- and middle-income countries [1]. COPD is the third most common cause of 30-day readmissions in the United States, with annual healthcare costs reaching USD 50 billion. Up to 70% of COPD-related medical expenses are attributable to acute exacerbations [2]. Smoking (46.0%), ambient particulate matter pollution (20.7%), and occupational exposure to particulate matter, gasses, and fumes (15.6%) were the prior known factors that had the greatest impact on the disability-adjusted life years rates for COPD [3].

The relationship between COPD and mental health has recently attracted a lot of research attention, given its impact on quality of life [4]. COPD has a significant negative impact on patients’ mental health. Compared to the general population, people with COPD have greater rates of depression and anxiety. According to Atlantis et al., the relative risk of patients with COPD developing depression is 1.69 compared to those without COPD [5]. When compared to other chronic comorbid conditions, COPD patients may even have higher rates of both anxiety and depression [6]. In addition, dyspnea and anxiety disorders are closely related. Anxious people may experience dyspnea and exacerbation of underlying COPD symptoms without an intrinsic reason, frequently in the context of panic disorder and agoraphobia leading to increased hospitalizations [7]. Depending on the population questioned and the instruments employed to measure depression and anxiety, the stated prevalence of these conditions vary widely. In this up-to-date narrative review, we aim to describe the association between COPD, anxiety, and depression, and highlight the various methods for screening and managing COPD.

## 2. Epidemiology and Prevalence

The global prevalence of COPD in people of age 30–79 years was 10.3% or 391.9 million people in 2019 using the GOLD criteria [8]. The frequency of depression varies greatly among patients with stable COPD in a primary care setting ranging, from 10% to 57%, while the prevalence of anxiety varies widely, between 7% and 50% [6]. The variation stems from the patient population under focus and the clinic setting. Some of the tools used in these studies were PRIME-MD (Primary Care Evaluation of Mental Disorders), Geriatric Mental State Schedule (GMS) screening questionnaire, Brief Assessment Schedule Depression Cards (BASDEC). The prevalence of depression was also consistently elevated in patients with COPD (RR, 1.69; 95% CI, 1.45–1.96) [5]. In a recent longitudinal study [9] of 35,000 patients with COPD and with a follow-up of 10 years, the incidence of depression was 16.2 cases per 1000 person-years in the COPD group compared with 9.4 cases per 1000 person-years in the non-COPD control group. In addition, those with severe COPD were twice as likely to develop depression compared with patients with mild COPD In patients with severe COPD (FEV1 <50% predicted), the prevalence of depression was 25.0% compared with 17.5% in controls and 19.6% in patients with mild to moderate COPD [10]. Prognosis in COPD is negatively impacted by depression and anxiety, which increases the chance of death and acute exacerbations. Atlantis et al. noted that depression or anxiety consistently elevated the risk of COPD adverse outcomes (RR, 1.43; 95% CI, 1.22–1.68). Comorbid depression increased mortality risk by 1.83 (95% CI, 1.00–3.36) and anxiety raised the risk of mortality by 1.27; (95% CI, 1.02–1.58). In COPD, poor health-related quality of life (HRQoL) is predicted by anxiety and depression. At 1-year follow-up, depression and poor HRQoL had a significant positive correlation (pooled r = 0.48, 95% confidence interval: 0.37–0.57, *p* < 0.001). Anxiety was also significantly correlated with worse HRQoL at 1-year follow-up (pooled r = 0.36, 95% confidence interval 0.23–0.48, *p* < 0.001) [11].

## 3. Pathophysiology

The mechanism of depression and anxiety in COPD is still not completely understood, as the relationship is complex [6]. The biological mechanism between COPD and depression is still unknown. Interestingly, these two disorders are considered heritable, and there is recent debate on whether to consider genetic causes for the association seen between COPD and depression or anxiety. The estimated genetic heritability for COPD is 25–37%, and that of Major Depressive Disorder (MDD) is 28–51%. Forced expiratory volume in one second (FEV1) and forced vital capacity (FVC) are also heritable factors that have an estimated heritability range of 18 to 50% [12]. One possible suspected mechanism relating depression and COPD is the “overspill” theory, where it is suspected that inflammatory markers spill over into the general circulation causing systemic inflammation. In this light, markers such as sTNFR-1 (soluble tumor necrosis factor alpha receptor-1) has shown a strong association with depression rates in patients with COPD [6]. One study showed a positive relationship between CRP levels in COPD patients and depression when compared to patients without depression. This indicates that CRP could also be a risk factor for depression [13]. The other proposed mechanism is smoking and hypoxemia, which also affects mental health in COPD patients. Periventricular white matter lesions are associated with hypoxia and are sometimes seen in patients with depression and COPD [6]. Anxiety and panic attack symptoms are also seen in patients with COPD especially those with acute exacerbations. The relationship between CO2/H+ sensitive neurons involved ventilation mechanisms and their role in increasing dyspnea has been implicated in anxiety and in panic attack symptoms. COPD patients with persistent hypercarbia are at an increased risk of such dyspneic spells and become more susceptible to anxiety attacks [6].

**Figure 1:**
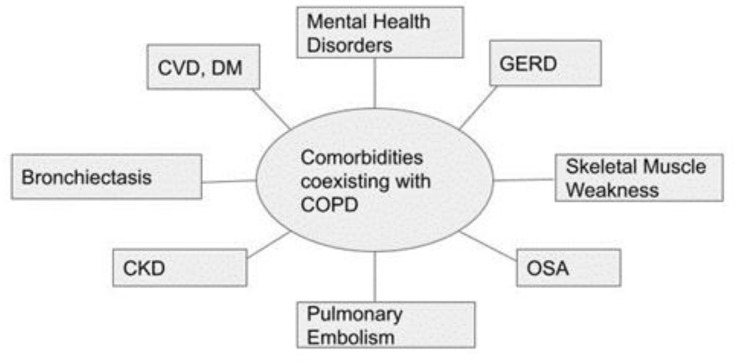
Clinical comorbidities coexisting with COPD [17,18]. Abbreviations: CKD, chronic kidney disease; CVD, cardiovascular disease; COPD, chronic obstructive pulmonary disease; DM, diabetes mellitus; GERD, gastroesophageal reflux disease; OSA, obstructive sleep apnea.

Multiple comorbid conditions sometimes co-exist in patients with COPD (Figure 1). Mental health disorders are an underrecognized factor in patients with COPD. Mental health disorders significantly cause increased disability in older people as they also affect quality of life [14]. Chronic stress weakens the immune system, increasing susceptibility to respiratory infections and acute exacerbations. Even though both anxiety and depression sometimes coexist, depression was found to be a stronger predictor of readmissions [15]. This was thought to be related to increasing exacerbations from medication non-adherence and deteriorating socio-economic conditions, which often affect patients with depression [16].

Other than socio-demographics, education, income, gender, perceived quality of life, disease severity, airflow obstruction, exercise scores (BODE), persistent smoking, long-term oxygen therapy, previous admissions, sense of loss, and inability to cope all have an influence [13,16]. In addition, low self- esteem, sexual abuse in childhood, family history of depression, disturbed family situation, traumatic experiences, also increase the risk of anxiety and depression [19]. Every time there is an exacerbation and subsequent hospitalization, it leads to a vicious cycle of worsening depression or anxiety, subsequently increasing the risk of deteriorating respiratory health [16]. Depression and anxiety are now considered modifiable factors impairing quality of life in patients with COPD. Addressing external factors such as medication noncompliance and accessibility to pulmonary rehabilitation programs can break the cycle of such exacerbations and re-admissions improving the prognosis of such patients [15].

## 4. Clinical Features

Patients with COPD frequently have symptoms of dyspnea, cough, and generalized fatigue. In addition, they may experience varying severity of depression symptoms (irritability, tearfulness, brooding, obsessive ruminations, phobia, and excessive worry over their physical health). These may range from short-term sub-clinical depressive symptoms to dysthymia (long-term chronic symptoms that are not disabling) to clinical depression [20]. It has been noted that patients with COPD and depression experience more dyspnea than their counterparts without depression. This was demonstrated by a study of 836 patients in whom greater dyspnea was noted in depressed vs. no-depression COPD patients (MRC dyspnea scale, 2.07 vs. 1.32; *p* <0.0001) [21]. This may also be attributed to the fact that these patients continued to smoke tobacco [22]. Tobacco smoke is considered an important environmental risk factor for the development of COPD, and high levels of anxiety is in turn a risk factor for smoking initiation [23,24]. Therefore, it is likely that patients with COPD secondary to smoking tobacco have higher levels of anxiety as well. The presence of depression and anxiety in patients with COPD has an effect on ‘vital exhaustion’ (fatigue and lack of energy, worsening irritability, and feelings of demoralization), which leads to poor health status [25].

## 5. Clinical Implication of Anxiety and Depression on Patients with COPD

### 5.1. Impact on Acute Exacerbation of COPD

Acute Exacerbation of COPD (AECOPD) has well-established acute and long-term adverse effects on health status beyond pulmonary function [26]. A recent study has noted depression to be an independent factor for AECOPD, with a higher risk of readmission for AECOPD (OR 2.06, 95% CI 1.28; 3.31), regardless of lung function and severe exacerbations in the previous year [27]. Similarly, a systematic review concurred high re-admission rates in patients with depression hospitalized for AECOPD [28], as did a retrospective study noting an association between depression and readmissions evaluated at 30, 90, and 365 days [29]. 

The association between anxiety and risk for AECOPD has shown variability in studies, with some denoting no association [27,30], while data from an observational study notes a higher risk of AECOPD with anxiety [31]. The differences in these results are likely from various methods used for the assessment of anxiety and depression as well as the heterogeneity of populations. It has been shown in a meta-analysis that COPD patients with anxiety are at greater risk for exacerbations requiring treatment in the community, whereas those with depression were found to be at higher risk for exacerbations requiring in-patient treatment [30].

### 5.2. Impact on Mortality

Studies have noted that depressive symptoms are associated with an increased risk of mortality both in the hospital and the outpatient setting [32,33,34,35]. A similar association with anxiety is not as clear, with fewer studies depicting increased mortality [33,36], while one study denotes the mortality benefit of anxiety in hospitalized COPD patients [37]. There may be a component of medication non-adherence in COPD patients with anxiety leading to increased mortality, as well as anxiety itself being a clinical marker for disease severity in this group. It is possible that the earlier management through hospitalization of COPD patients with anxiety might have led to improved mortality as these patients report more severe symptoms. Comorbid anxiety and depression are associated with an increased risk of mortality in COPD patients, with relative risks of 2.29 and 1.27, respectively [5]. This increase in mortality risk has also been noted post hospital discharge [28,38].

### 5.3. Impact on Medication Adherence

A meta-analysis has shown that patients with depression and anxiety symptoms are three times more likely to be non-adherent to their prescribed medications [39]. This holds true for COPD patients with depression and anxiety as well [40]. The impact of nonadherence to COPD therapies leads to higher hospitalization rates, costs, as well as increased emergency department visits [41,42].

### 5.4. Impact on Pulmonary Rehabilitaion 

The clinical trial results, including 238 volunteers, revealed that the negative impact of anxiety and depression in COPD patients remained unchanged throughout pulmonary rehabilitation. That included significantly increased dyspnea, reduced functional performance, and quality of life. These results show the importance of diagnosing and treating anxiety and depression in patients with COPD [43]. 

### 5.5. Impact on Quality of Life

COPD, like many other chronic illnesses, negatively impacts quality of life. The presence of depression and anxiety among COPD patients further deteriorates quality of life and has been noted to have the strongest correlations with self-reported health status and reduced health-related quality of life (HRQoL) [44]. One study noted that COPD patients’ quality of life was correlated more with the presence of depressive symptoms than with the severity of COPD as measured by FEV1 values [45]. Depression adversely affects physical functioning and has been attributed to causing about 18% variance in physical functioning in COPD patients [46]. The increased dyspnea experienced by patients with COPD and depression indeed leads to social isolation and worsening physical inactivity leading to further deconditioning [47]. COPD patients with depression and anxiety experience low self-esteem, high apathy, and high denial of impulse life [48]. Depression also impacts their end-of-life decisions and negatively affects their relationships [49].

## 6. Screening and Diagnosis

Anxiety and depression are underdiagnosed in COPD patients. This is largely secondary to the overlapping of somatic symptoms of depression with symptoms of COPD [50]. The Global Initiative for Chronic Obstructive Lung Disease (GOLD) guidelines recommend that new COPD patients should have a detailed medical history, including for depression and anxiety [51]. Additionally, COPD assessment tools like COPD Assessment Test and the COPD Clinical Questionnaire have incorporated questions indicative of symptoms of depression and anxiety [52]. All newly diagnosed COPD patients should have a frequent assessment of depression and anxiety using medical history and clinically validated screening tools. These assessments should be performed at follow-up visits/annual visits, hospital discharge, and referral to pulmonary rehabilitation.

The following validated screening tools can be used to assess depressive and anxiety symptoms in COPD patients.

- Patient Health Questionnaire-2, 9 (PHQ -2 and 9): widely used with 2 and 9 item versions [53].
- Beck Depression Inventory (BDI): 21-item self-reported questionnaire [54].
- Geriatric Depression scale (GDS) [55].
- Centre for Epidemiological studies scale on Depression (CES-D): score of 16 or higher on the CES-D is considered a possible case for depression [56].
- Hospital and Anxiety Depression Scale (HADS) [57].
- Brief Assessment Schedule Depression Cards (BASDEC) [45].
- Anxiety Inventory for Respiratory (AIR) Disease scale [58,59].
- COPD Anxiety Questionnaire (CAF): early identification of COPD-related anxiety [60].
- Primary Care Evaluation of Mental Disorders (PRIME-MD): comprises 26 yes or no questions on the five most common psychiatric disorders, including depression and anxiety [61,62].
- Patient Health Questionnaire-3 (PHQ-3) [61].
- Generalized Anxiety Disorder 7-item (GAD-7) scale: scores seven common anxiety symptoms [63].
- General Health Questionnaire-version 20 (GHQ-20) [57].
- Beck Anxiety Inventory (BAI): 21-item self-report questionnaire [54].

**Figure 2:**
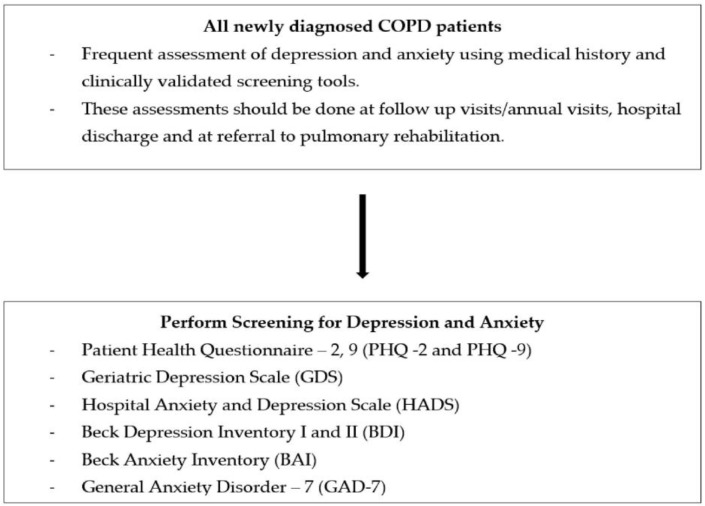
Proposed algorithm for assessing depression and anxiety in patients with COPD.

All of the above screening tests can be used with no consensus if one of them is more consistent than the other for diagnosing anxiety and depression in patients with COPD [64]. The gold standard for the diagnosis of major depressive disorder or anxiety (generalized anxiety disorder, panic attacks, panic disorder) is determined by the DSM-IV criteria, as described in Figure 2, which is performed by a psychiatrist or a clinical psychologist through structured interviews.

## 7. Management

Managing anxiety and depression should be a multidisciplinary approach to provide the most appropriate form of therapy to COPD patients. Communication and eliciting a clear past medical history are key in choosing the necessary treatment intervention [65]. Anxiety and depression in COPD patients can be managed pharmacologically and non-pharmacologically in order to attain a holistic approach [14].

Two studies have compared psychological therapies plus a co-intervention versus the co-intervention alone (i.e., pulmonary rehabilitation (PR)). The results suggest that psychological therapy combined with a PR program can reduce depressive symptoms more than a PR program alone (SMD 0.37, 95% CI −0.00 to 0.74; *p* = 0.05; 2 studies, 112 participants) [66].

Sixty-five Randomized Control Trials of intervention of pulmonary rehabilitation in patients with COPD involving 3822 participants for inclusion in the meta-analysis where the health-related quality of life (HRQoL) and/or functional (FEC) or maximal (MEC) exercise capacity were measured showed that the pulmonary rehabilitation group resulted in the mean forced expiratory volume at one second (FEV1) of 39.2% predicted, and for the conventional care group, it was 36.4%. Clinically significant improvements were seen in the degree of dyspnea and fatigue, and emotional function and gave participants the sense of control they had over their diagnosis [67].

One randomized and well-controlled trial with adequate sample size and application of DSM diagnostic criteria reported significant improvements in depressive symptoms after Cognitive Behavioral Therapy, which was sustained until 8-month follow-up, but not after enhanced standard care. Studies have found a single two-hour session of CBT to reduce depressive symptoms in mild depressed COPD patient [68]. A recent meta-analysis included 16 randomized controlled trials and found significant improvements in anxiety, depression, quality of life, and emergency room visits in COPD patients treated with CBT. However, fatigue, exercise capacity, self-efficacy, and sleep quality were not impacted [69]. Psychological and mind-body therapies have a promising role as well. A recent Cochrane systematic review studied the effectiveness of psychological therapies for treating depression in moderate–severe COPD patients. Compared to no-intervention or education, psychological therapies improved depressive symptoms. Combining psychological therapies with a co-intervention is beneficial, as well. Psychological therapies also improved quality of life and showed significant reduction in hospital admission rates [66]. Another Cochrane systematic review included three prospective randomized controlled trial to assess the effectiveness of psychological therapies with a co-intervention vs. co-intervention alone in COPD patients with anxiety. Even though the quality of evidence was low, there were improvements in anxiety scores over 3 to 12 months when combining psychological therapies with a co-intervention [70]. A more recent meta-analysis with over 3000 patients studied the psychological and physical impact of psychosocial intervention in COPD patients. it found small but statistically significant effects of psychosocial interventions on combined psychological and physical outcomes. Interestingly, older age and longer duration of interventions were associated with smaller effects on psychological outcomes [71].

The pharmacologic options for treating depression and anxiety in COPD patients are tricyclic antidepressants (TCAs), selective serotonin reuptake inhibitors (SSRIs), serotonin and noradrenaline reuptake inhibitors (SNRI), noradrenergic and specific serotonergic antidepressants, norepinephrine, and dopamine reuptake inhibitors, and melatonergic antidepressants [72]. In a few randomized, double-blind, placebo-controlled studies, sertraline, fluoxetine, citalopram, and paroxetine caused improvements in quality of life, dyspnea, and fatigue [73]. Caution is advised when using these medications in older patients. In a retrospective analysis, the use of SSRI/SNRI in older adults was associated with higher rates of hospitalization for COPD or pneumonia, COPD or pneumonia-related and all-cause mortality [74]. Table 1 and Table 2 enlist non-pharmacological and pharmacological interventions for the management of anxiety and depression in patients with COPD, respectively. Table 3 enlist ongoing trials comparing interventions for managing anxiety and depression in patients with COPD, respectively. These trials were selected from the national clinical trials database at www.clinicaltrials.gov (accessed on 1 February 2023).

## 8. Conclusions

COPD patients with comorbid anxiety or depression experience more acute exacerbations and incidences of rehospitalization within a 12-month period. They also carry a higher risk of mortality than COPD patients without these comorbidities. COPD patients with anxiety or depression benefit from pulmonary rehabilitation, CBT, and cautious use of antidepressants. Active investigation and research are necessary for adequate and effective screening, diagnosis, and management of anxiety and depression in COPD patients to decrease their negative impact on quality of life and to reduce readmission and mortality rates in this population.

## Figures and Tables

**Table 1:** Non-pharmacologic interventions [14,65].

Non-Pharmacologic Interventions
Cognitive Behavioral Therapy (CBT) Exercise
Education
Relaxation techniques (Breathing exercises, sequential muscle relaxation, hypnosis, mindfulness meditation)
Home-based psycho-educational intervention using telephone health mentoring
Lifestyle modification (smoking cessation, nutritional foods, quality sleep and physical exercise)
Multidisciplinary pulmonary rehabilitation
Yoga
Counseling
Collaborative care model
Social support and respite care for caregivers

**Table 2:** Pharmacologic interventions [14,65,75].

Pharmacologic Interventions
Norepinephrine and dopamine-reuptake inhibitor: Bupropion
Norepinephrine and serotonin modulator: Mirtazapine
Atypical antipsychotics
Tricyclic antidepressants: Nortriptyline
Selective Serotonin-reuptake inhibitors (SSRIs)

**Table 3:** Ongoing trials.

NCT Identifier and/or Author	Study Design	Intervention	Primary Outcome Measurement Tool
NCT04868357	Trial: HYPNOBPCO_2; 2-arm, cluster-randomized, statistician-blinded superiority monocenter trial, 100 participants with Hypnosis” (treatment) and “Relaxation” (active control). “Hypnosis” will consist Pulmonary Rehabilitation Program, supplemented by two educational sessions for teaching self-hypnosis. Relaxation group will be identical, except standard relaxation exercises will be taught instead of hypnosis.	Hypnosis as a tool to manage anxiety and dyspnea post pulmonary rehabilitation program (PRP).	State-Trait Anxiety Inventory (STAI-6) Hospital Anxiety and Depression inventory (HADS) Multidimensional Dyspnea Profile (MDP)
NCT05506202	Randomized clinical trial; n = 36	Treatment group: Basic body awareness therapy Control group: basic and advanced respiratory physiotherapy interventions	Modified Medical Research Council scale for breathlessness Dyspnoea-12 (Chinese version) St. George’s Respiratory Questionnaire (Chinese version) COPD Self-Efficacy Scale (CSES) (Chinese version) 6 min walking test
NCT04860375	Prospective, open label, cohort study; n = 110	Treatment group: Multidisciplinary patient care (dietary program, adjusted exercise program, psychological counseling, treatment of comorbidities) Control group: Standard care	Total number of hospitalizations
NCT04898972	Randomized clinical trial; n = 80	Mindfulness-based stress reduction (body scan; sitting meditation; awareness movement exercises; and walking meditation) Control group: informative booklet on stress reduction strategies	Perceived Stress Scale (PSS) Generalized Anxiety Disorder scale (GAD-7) Patient Health Questionnaire (PHQ-9)

## Data Availability

The data supporting this research are available from the corresponding author on reasonable request.
